# Evaluation der Mikrozirkulation bei kritisch kranken Patienten: Relevanz, praktische Möglichkeiten und wissenschaftliche Evidenz

**DOI:** 10.1007/s00101-020-00832-4

**Published:** 2020-08-14

**Authors:** J. Wollborn, C. Jung, U. Göbel, R. R. Bruno

**Affiliations:** 1 Klinik für Anästhesiologie und Intensivmedizin, Universitätsklinikum Freiburg, Medizinische Fakultät, Albert-Ludwigs-Universität Freiburg, Hugstetter Str. 55, 79106 Freiburg, Deutschland; 2 Klinik für Kardiologie, Pneumologie und Angiologie, Universitätsklinikum Düsseldorf, Düsseldorf, Deutschland; 3 Klinik für Anästhesiologie und operative Intensivmedizin, St. Franziskus-Hospital Münster, Münster, Deutschland

**Keywords:** Mikrozirkulation, Organdysfunktion, Kritisch kranke Patienten, Intensivmedizin, Individualisierte Medizin, Microcirculation, Organ dysfuntion, Critically ill patients, Intensive care medicine, Individualized medicine

## Abstract

Für die Aufrechterhaltung der Organdurchblutung ist eine intakte Makro- und Mikrozirkulation essentiell. Sowohl das wachsende Verständnis um die Bedeutung der Mikrozirkulation im Organversagen als auch die Möglichkeit, diese zu visualisieren, lenken die Aufmerksamkeit der Intensivmedizin auf die mikrovaskuläre Endstrombahn. Als Surrogat-Parameter sind die Rekapillarisierungszeit, der „mottling score“ und die Messung des Serum-Laktats bereits lange in der klinischen Praxis etabliert. Neuere Studien messen der Echtzeit-Darstellung der sublingualen Mikrozirkulation mittels Intravital-Videomikroskopie eine immer größere Bedeutung bei. Verschiedene Studien unterstreichen hierbei die Mikrozirkulation als prognostischen Parameter. Darüber hinaus ermöglicht die Erhebung von objektivierbaren Messwerten, diese in der Zukunft zur individuellen Therapiesteuerung weitergehend zu untersuchen.

## Abstract (en)

As one critical parameter for organ perfusion, microcirculation and its monitoring are gaining increasing attention for modern intensive care medicine. The growing understanding of its importance in organ failure and the improved modes of its visualization mark microcirculation as an interesting target. Surrogate parameters for organ perfusion, like re-capillarization (“Recap”) time, the “mottling score” or the measurement of serum lactate have long been established in clinical practice. A growing body of evidence is hinting towards online visualization of sublingual microcirculation using intravital video microscopy, which was shown to be of prognostic value. Furthermore, the measurement of objective and reproducible parameters hint towards use in individualized hemodynamic therapy.

## Einleitung und Physiologie der Mikrozirkulation

Für die Organperfusion ist sowohl eine intakte Makro- als auch Mikrozirkulation notwendig. In der Praxis werden häufig makrozirkulatorische Parameter als therapeutische Ziele definiert, wie z. B. die „Surviving Sepsis Campaign Guidelines“, die einen mittleren arteriellen Zielblutdruck von 65 mm Hg vorschlagen [1]. Ausschlaggebend dafür ist die Annahme, dass eine adäquate Makrozirkulation gleichbedeutend mit einer guten Endorgandurchblutung ist. Jedoch kann bei kritisch Kranken der Verlust der „hämodynamischen Kohärenz“ eintreten [2]. Eine Steigerung des arteriellen Blutdrucks korreliert dann nur sehr eingeschränkt mit einer verbesserten Organperfusion [3]. Dies könnte die negativen Ergebnisse der letzten „Early-goal-directed-therapy“-Studien erklären [4]. Daher stellt sich die Frage, ob die Mikrozirkulation als Zielparameter nicht besser geeignet wäre [5].

Laminare Flusseigenschaften unterliegen verschiedenen Einflussfaktoren wie Gefäßdiameter und -länge, Viskosität und Druckdifferenz (Hagen-Poiseuille-Gesetz). Die Mikrozirkulation ist verantwortlich für eine adäquate Sauerstoff‑, Flüssigkeits- und Nährstoffversorgung sowie den Abtransport toxisch-zellulärer Abfallprodukte aus den jeweiligen Zellverbänden [6]. Anatomisch gliedert sie sich in Arteriolen, Kapillaren und Venulen. Die Arteriolen haben einen Gefäßdiameter unter 100 µm. Sie reagieren schnell auf autonome Stimuli und Mediatoren, um den Gefäßdurchmesser flexibel anzupassen. Die Kapillaren haben einen maximalen Diameter von 10 µm ohne wesentliche Vasomotorik [7]. In diesem Kompartiment determinierten verschiedene Parameter den für die Organfunktion entscheidenden Stoffaustausch: Die Dichte an perfundierten Gefäßen und die jeweilige Strecke (evtl. vergrößert durch Ödem) zwischen Gefäß und Zelle bestimmen die Diffusion, während die Rheologie mit der Blutviskosität und den Fließeigenschaften die Konvektion beeinflusst [5]. Infobox 1 gibt einen Überblick zu verschiedenen Einflussfaktoren auf die Mikrozirkulation. Venulen weisen einen Gefäßdiameter von 10 bis 100 µm auf und besitzen eine hohe Gefäßpermeabilität mit der Möglichkeit zur Diapedese von immunkompetenten Zellen (z. B. Leukozyten).

### Infobox 1 Einflussfaktoren auf die Mikrozirkulation^a^

- Herz-Zeit-Volumen
- Volumenstatus
- Viskosität des zirkulierenden Volumens
- Änderung der Gefäßpermeabilität
- Regionale Unterschiede in der Perfusion (z. B. AV-Shunts)
- Freisetzung der vasoaktiven Mediatoren
- Änderung der Gefäßinnervation
- Änderung der vaskulären Resistenz
- Änderungen des pH-Werts

^a^(Modifiziert und ergänzt nach [11])

## Bedeutung der Mikrozirkulation bei kritisch Kranken

Der Schock ist die „Extremform der Mikrozirkulationsstörung“. Die Mikrozirkulationsstörung korreliert frühzeitig und unabhängig mit der Prognose [8]. Pathophysiologisch können verschiedene Parameter der mikrovaskulären Endstrecke gestört sein: Ödeme beeinträchtigen die Diffusion signifikant [9]. Eine Anämie oder Hypovolämie kann durch den verminderten Anteil an Sauerstoffträgern sowohl die Diffusion (passiver Ausgleich von Konzentrationsunterschieden durch Teilchenbewegung) als auch die Konvektion (Transport durch Druckunterschiede) beeinträchtigten. Darüber hinaus beobachtet man bei der Sepsis häufig ein heterogenes Flussprofil, das zu funktionellen Links-rechts-Shunts führt [10]. Weiterhin ist auch die Dichte der Kapillaren für die Organperfusion wichtig, während eine verminderte kardiale Kontraktilität bzw. eine Hypotension die Fließeigenschaften auf Mikrozirkulationsebene negativ beeinflussen können [11]. Die Aspekte der reduzierten Verformbarkeit von Erythrozyten, die „Geldrollenbildung“ (auch „Rouleau-Phänomen“) und die Aktivierung der Endotheloberfläche spielen ebenso eine Rolle.

Die Intensivmedizin kann Teile dieser Parameter aktiv beeinflussen: Bei Hypovolämie kann die Flüssigkeitsgabe die Gewebeperfusion beeinflussen, während ein Flüssigkeitsentzug bei Hypervolämie das Gewebsödem und die konsekutive Verlängerung der Diffusionsstrecke reduzieren kann. Die Volumentherapie ist eine komplexe Stellgröße, die eine ausreichende Gewebeperfusion ohne Gewebsödem sicherstellen muss. Manifestationsarten von Mikrozirkulationsstörungen unterscheiden sich organabhängig: In der Lunge kann die Rekrutierung von arteriovenösen Shunts zum Aggravieren einer Hypoxämie führen. Die akute Nierenschädigung kann einer gestörten Gewebsperfusion zugrunde liegen [12, 13]. Mikrozirkulationsstörungen können auch die Funktion aller anderen Organe wie der Leber, des Gastrointestinaltrakts, des Zentralnervensystems, des Knochenmarks und nicht zuletzt des Herzens empfindlich stören. Entscheidend ist, dass bereits einzelne Organschädigungen die Prognose eines kritisch Kranken einschränken. Es ist daher entscheidend, Organdysfunktionen und zugrunde liegende Mikrozirkulationsstörungen als potenziell reversible Ursache frühzeitig zu erkennen.

## Beurteilung – klinisch, laborchemisch und apparativ

Zur Beurteilung der Mikrozirkulation existiert eine große Bandbreite an qualitativen und quantitativen Verfahren (Tab. 1). Einen großen Stellenwert hat dabei die kapillare Wiederfüllungszeit (Rekapillarisierungszeit, „Nagelbettprobe“ oder „capillary refill time“). Diese Untersuchung ist schnell und ohne apparativen Aufwand möglich. Dabei wird der Fingernagel für eine kurze Zeit bis zur Weißfärbung ins Nagelbett gedrückt. Beim Loslassen des Drucks färbt sich das Nagelbett als Folge der zurückkehrenden Durchblutung rasch (unter 2 s) wieder rot. Korrekt durchgeführt kann sie das Vorliegen einer relevanten Mikrozirkulationsstörung detektieren. Es besteht jedoch eine erhebliche Untersucherabhängigkeit [14]. Die Rekapillarisierungszeit kann als früher Zielparameter für die Volumentherapie bei Sepsispatienten fungieren [15]. Eine weitere klinische Möglichkeit ist der „Mottling Score“. Dieser beschreibt das Ausmaß der Hautmarmorierung in Relation zum Knie. Der „Mottling Score“ ist ein unabhängiger starker Prädiktor für die Prognose septischer Patienten [16].

**Table 1:** 

Methode	Vorteile	Limitationen
Rekapillarisierungszeit	Sensitiv; sofort einsetzbar	Untersucherabhängig; erschwerte Quantifizierung
„Mottling Score“ [16]	Sensitiv; sofort einsetzbar	Untersucherabhängig
Lactat	Sehr sensitiv; vergleichbar; überall verfügbar	Zeitabhängig; viele Einflussfaktoren
Mitochondriales pO_2_	Sensitiv; nichtinvasiv; objektivierbar, erfasst O_2_-Verwertungsstörungen	In klinischer Erprobung; es gibt keine Aussage über Flusseigenschaften der Mikrozirkulation
SDF-/IDF‐Intravitalmikroskopie	Sensitiv; nichtinvasiv, verschiedene Aspekte der Mikrozirkulation sind objektivierbar, nach Erlernen der Technik hohe Reliabilität	Teuer; untersucherabhängig

*SDF* „sidestream-darkfield imaging“, *IDF* „incident-darkfield imaging“

Aktuell stellt das Lactat den häufigsten quantifizierten Surrogatparameter für die Mikrozirkulation dar. Sowohl eine Einzelwertbestimmung des Lactats als auch die Clearance-Kinetik haben Eingang in die Leitlinien gefunden [1]. Von Bedeutung ist, dass das Lactat zahlreichen Einflussfaktoren wie z. B. der Leberfunktion unterliegt [17]. Insgesamt gilt das Lactat als sehr sensitiv, aber wenig spezifisch für das Vorliegen einer Mikrozirkulationsstörung. Eine weitere Limitation ist, dass ein erhöhtes Lactat die Folge einer bereits manifesten Mikrozirkulationsstörung darstellt. Aus diesem Grund wurden zahlreiche Versuche unternommen, um eine Mikrozirkulationsstörung bereits vor Entwicklung einer Sauerstoffschuld im Gewebe zu detektieren.

Eine neuere Methode, das Sauerstoffangebot und die -verwertung zu beurteilen, stellt die Messung des mitochondrialen pO_2_ (p_mito_O_2_) dar [18]. Hierbei kann nichtinvasiv und transkutan über die fluoreszierenden Eigenschaften der Enzyme der Atmungskette (in den Mitochondrien) auf die Sauerstoffverwertung rückgeschlossen werden. Ein erstes CE-zertifiziertes System zur bettseitigen Messung des p_mito_O_2_ findet sich aktuell in klinischer Erprobung.

**Figure 1:**
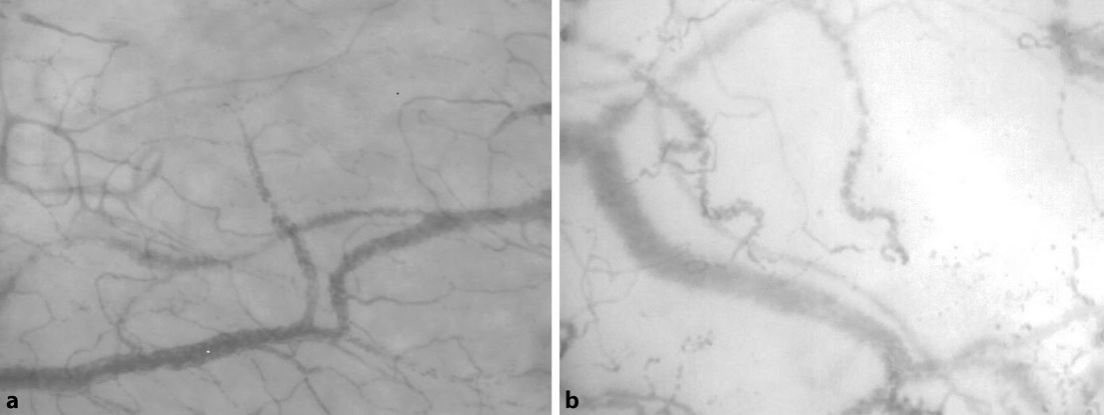

Daneben gewinnt die direkte und objektivierbare Visualisierung der Mikrozirkulation stetig an klinischer Bedeutung [9]. Diese ermöglicht Rückschlüsse auf verschiedene Eigenschaften des Endstromgebiets: Indirekte Messmethoden wie die Gewebekapnometrie (im Magen), die Laser-Doppler-Flowmetrie und die HbO_2_-Spektroskopie konnten sich hierbei aber nicht klinisch durchsetzen. Einen Durchbruch brachte hingegen die Entwicklung der bettseitigen Intravitalvideomikroskope „sidestream-darkfield“ (SDF, z. B. MicroScan^TM^; Fa. Microvision Medical, Niederlande) und Incident-Darkfield (IDF, z. B. CytoCam; Fa. Braedius Medical, Niederlande). Diese können durch Illumination und Reflexion des Gewebes das mikrovaskuläre Gefäßbett in hoher Auflösung darstellen (Abb. 1) und werden vornehmlich sublingual angewendet. Die Messung ist einfach zu erlernen und kann beliebig oft in Echtzeit wiederholt werden, während sie u. a. eine Veränderung des Lactats antizipiert [11].

Einen weiteren Durchbruch in der Anwendung dieser Systeme bedeutete die bettseitige semiautomatische Auswertung, die ein direktes und schnelles Feedback erlaubt. Durch die Zunahme des klinischen Interesses an der Mikrozirkulation wurde nun bereits eine zweite, aktualisierte Konsensusempfehlung der European Society of Intensive Care Medicine (ESICM) zur standardisierten Erfassung und Beurteilung der sublingualen Mikrozirkulation erarbeitet [9].

## Ausblick

Die Visualisierung der sublingualen Mikrozirkulation ist ein mögliches Screeninginstrument zur Abschätzung der Prognose des kritisch Kranken. Unter anderem konnte für den kardiogenen Schock, die Sepsis und nach stattgehabter kardiopulmonaler Reanimation eine Relevanz hinsichtlich des Patienten-Outcome gezeigt werden [11]. Auch bei der extrakorporalen Membranoxygenierung (ECMO) kann die Messung der Mikrozirkulation zur frühen Prognostizierung genutzt werden [18] und konnte in Studien bereits beim Weaning von der ECMO unterstützen [19, 20]. Bei Anämie könnte die Mikrozirkulation einen „physiologischen“ Zielparameter darstellen und – unabhängig vom Hämoglobinwert – helfen, Patienten, die von einer Transfusion profitieren, zu identifizieren [21]. Die schnelle bettseitige Erhebung von objektivierbaren Parametern und die Durchführung von Scores, wie dem Point of Care Microcirculation (POEM) Score mit geringer untersucherabhängiger Variabilität [22], sind dabei von höchster Bedeutung. Um die sublinguale Mikrozirkulation jenseits von Studien als Zielparameter zu nutzen, fehlt es allerdings noch an prospektiven randomisierten Studien.

Die Zukunft der Intensivmedizin kann in einer „personalisierten physiologischen Medizin“ gesehen werden [5]. Ein zentraler Aspekt könnte die individuelle Messung der Organperfusion sein. Weitere Studien, die die Steuerung der Volumentherapie, vasoaktiver Substanzen, von Blutprodukten und additiven Therapieverfahren mittels Messung der Mikrozirkulation zielgerichtet ermöglicht, werden mit Spannung erwartet (z. B. die Untersuchung der Mikrozirkulation als Parameter der Therapiesteuerung bei Patienten im Schock, NCT04173221).

## Fazit für die Praxis

Die Mikrozirkulation ist ein entscheidender Parameter für die Organperfusion und könnte ein weiterer Parameter sein, um eine Prognose abzuschätzen. Es erscheint daher logisch, dass die direkte, nichtinvasive Mikrozirkulationsmessung für die klinische intensivmedizinische Praxis an Bedeutung gewinnen wird. Sie hat das Potenzial, sowohl gefährdete Patienten zu identifizieren als auch deren individuellen Therapieziele zu überwachen.
